# The effects of fampridine on MS-related fatigue: a systematic review

**DOI:** 10.3389/fneur.2025.1720316

**Published:** 2026-01-19

**Authors:** Mohsen Rastkar, Christian Cordano, Mahsa Ghajarzadeh, Bardia Nourbakhsh

**Affiliations:** 1 Student’s Scientific Research Center, Tehran University of Medical Sciences, Tehran, Iran; 2 UCSF Weill Institute for Neurosciences, Department of Neurology, University of California San Francisco, San Francisco, CA, United States; 3 Department of Neurology, Johns Hopkins University School of Medicine, Baltimore, MD, United States

**Keywords:** dalfampridine, fampridine, fatigue, multiple sclerosis, systematic review

## Abstract

**Background:**

Slow-release 4-aminopyridine (fampridine) has been shown to improve walking function in people with multiple sclerosis (MS). Its effect on other MS symptoms, such as fatigue, remains controversial. We performed this systematic review to summarize the evidence of the effect of fampridine on fatigue in patients with MS.

**Methods:**

PubMed, Scopus, EMBASE, Web of Science, google scholar, and ProQuest were searched for randomized trials or observational studies reporting fatigue scores before and after the treatment with fampridine. We summarized the findings of all relevant reports.

**Results:**

A literature search revealed 2,675 records; after removing duplicates, we had 1,504 records. Ninety-seven full texts were evaluated, and finally, 33 studies remained for systematic review. Most studies were done in USA, France, Germany, and Italy. The participants’ age and the duration of studies ranged between 39 and 54 years and 2 and 48 weeks, respectively. Out of 20 non-randomized or observational studies, 19 reported a benefit of fampridine in improving MS fatigue; however, only three out of 13 randomized, placebo-controlled studies showed that fampridine improved fatigue better than a placebo.

**Conclusion:**

Overwhelmingly positive results of fampridine on fatigue reported in non-randomized and observational studies are compatible with the placebo-responsiveness of fatigue in MS. Randomized, placebo-controlled studies have provided inconsistent results on the effects of fampridine on MS fatigue. Although it is possible that fatigue, at least in a subgroup of people with MS, might respond to fampridine, high-quality, placebo-controlled, blinded, randomized trials are needed to show the efficacy of this medication in improving MS fatigue.

## Introduction

Fatigue, defined as a subjective lack of physical or mental energy, is one of the most common symptoms in patients with multiple sclerosis (MS), affecting between 65 and 90% (1). For many patients, fatigue affects the quality of life even more than physical disability (2).

Most patients report that fatigue interferes with their personal and professional life, leading to lower health-related quality of life and unemployment (3). Physical exercise and cognitive behavioral therapy are effective in improving MS fatigue, but commonly used pharmacotherapies, such as amantadine, modafinil, and psychostimulants, probably do not outperform placebo (4).

Fatigue in people with MS has a complex pathophysiology. Structural and functional changes in the central nervous system (CNS) and systemic and CNS inflammation play an important role (2, 3). Demyelination is the pathological hallmark of MS. Activity-dependent conduction block in chronically demyelinated axons has been proposed as one of the contributing mechanisms to MS fatigue (4). On the other hand, secondary fatigue is due to underlying medical or psychiatric conditions such as depression, breathing disorders, insomnia, sleep problems, and anxiety are common in patients with MS (1, 5). Secondary causes of fatigue, most notably breathing disorders and depression, are prevalent and operate through distinct mechanisms, but all exacerbate the subjective experience of fatigue and diminish quality of life (6).

Paranodal potassium channels that become redistributed in demyelinated axons contribute to conduction failure. Aminopyridines (including, slow release 4-aminopyridine) are potassium-channel blockers (7) acting by restoring conduction in demyelinated axons and enhancing transmission in normal myelinated axons by increasing calcium influx at presynaptic terminals (7). Slow-release 4-aminopyridine is known by several names, including dalfampridine, fampridine, and PR fampridine. We will use these terms interchangeably throughout this manuscript.

In 2010, the United States Food and Drug Administration (FDA) approved dalfampridine for walking improvement in patients with MS, and some studies have reported its positive effects on visual function, fatigue, cognition, gait, and balance in people with MS (8, 9).

Considering the disappointing results from the use of medications commonly prescribed to improve MS fatigue, there is a need for safe and effective pharmacotherapy for this common and disabling MS symptom. Several studies have evaluated the effects of fampridine on fatigue in patients with MS with mixed results. Currently, fampridine is not considered an option for treating fatigue in MS. We performed this systematic review to summarize the evidence of the effect of fampridine on fatigue in patients with MS.

## Methods

We followed Preferred Reporting Items for Systematic Reviews and Meta-Analyses (PRISMA) 2020 for reporting our systematic review (11).

### Eligibility criteria

*Inclusion criteria were:*

Randomized trials or observational studies reporting fatigue scores before and after the treatment with fampridine.

*Exclusion criteria were:*

Letters to the editor, case–control, and case reports studies. We also excluded studies that had no clear data regarding fatigue findings.

### Information sources

Two researchers of this study independently searched PubMed, Scopus, EMBASE, Web of Science, google scholar, and also conference abstracts and theses (ProQuest). They also manually checked the references of the included studies to prevent missing potential studies. The search was done on May 12^st^ 2024.

### Search strategy

The search strategy was designed using MeSH terms and relevant keywords as follows:

(((((((((((((((4-Aminopyridine[MeSH Terms]) OR (4-Aminopyridine[Text Word])) OR (4 Aminopyridine[Text Word])) OR (Dalfampridine[Text Word])) OR (Pymadine[Text Word])) OR (VMI-103[Text Word])) OR (VMI 103[Text Word])) OR (VMI103[Text Word])) OR (4-Aminopyridine Sustained Release[Text Word])) OR (4 Aminopyridine Sustained Release[Text Word])) OR (Sustained Release, 4-Aminopyridine[Text Word])) OR (Fampridine-SR[Text Word])) OR (Fampridine SR[Text Word])) OR (Fampyra[Text Word])) OR (Fampridine[Text Word])) AND (((((((Multiple Sclerosis[MeSH Terms]) OR (Multiple Sclerosis[Text Word])) OR (Sclerosis, Multiple[Text Word])) OR (Sclerosis, Disseminated[Text Word])) OR (Disseminated Sclerosis[Text Word])) OR (Acute Fulminating Multiple Sclerosis[Text Word])) OR (Multiple Sclerosis, Acute Fulminating[Text Word])).

### Selection process and collection

All search results were transferred to EndNote software, duplicates were deleted, and the title/abstract of potential studies were evaluated.

Two independent researchers evaluated the full texts of potential studies, and data were entered into Excel data sheets. In the case of discrepancy, a third researcher addressed the conflict.

### Data items

We extracted data regarding the total number of patients, first author, publication year, country of origin, mean age, sex, duration of disease, type of MS, Expanded Disability Status Scale (EDSS), and fatigue results.

### Study risk of bias assessment

We evaluated the risk of bias in clinical trials and cohort studies by ROBINS-I and ROBINS-II (10, 11).

## Results

**Figure 1:**
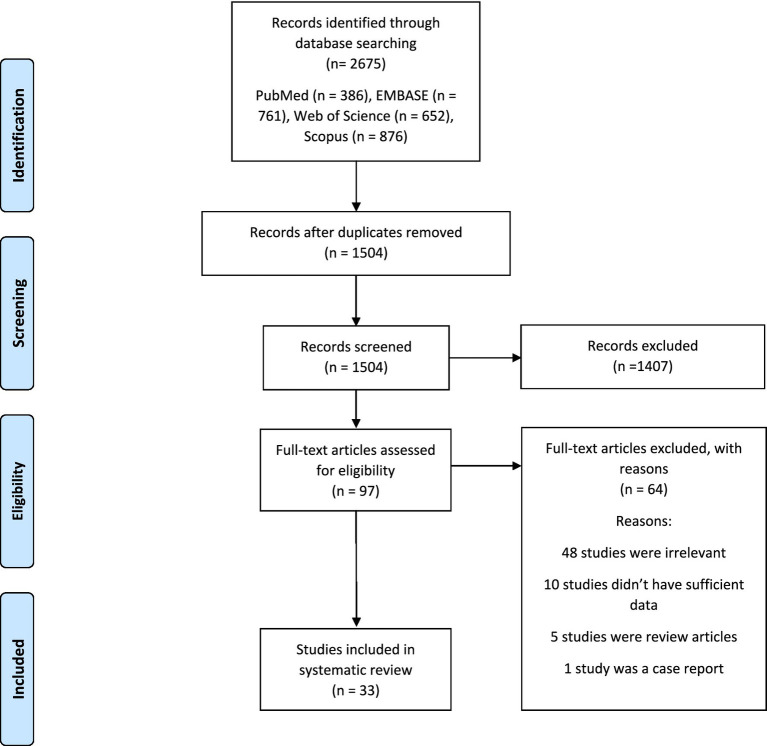
The flow chart of studies inclusion.

A literature search revealed 2,675 records; we had 1,504 records after removing duplicates. Ninety-seven full texts were evaluated, and finally, 33 studies remained for systematic review (Figure 1).

Nine conference abstracts were included. Most studies were done in the USA, France, Germany, and Italy. The age of participants and the duration of studies ranged between 32 and 55 years and 2 and 48 weeks, respectively.

Studies use various fatigue measures, including Modified Fatgiue Impact Scale (MFIS), Fatigue Impact Scale (FIS), Fatigue Severity Scale (FSS), visual analogue scale (VAS), Brief Fatigue Inventory (BFI), and Würzburg Fatigue Inventory for Multiple Sclerosis (WEIMuS) (Supplementary Tables 1, 2). There were 13 randomized, placebo-controlled studies and 20 non-randomized, uncontrolled, open-label, or observational studies. In placebo-controlled studies, the improvement in fatigue scores was compared between fampridine and placebo groups. In contrast, in uncontrolled studies, only the change in fatigue score after starting fampridine was compared to the scores before starting fampridine.

While only three out of 13 randomized, placebo-controlled blinded studies reported a statistically significant improvement in fatigue compared to the placebo group, 19 out of 20 non-randomized, uncontrolled observation studies reported improvement in fatigue after starting fampridine compared to before starting fampridine, at least in a subset of patients.

We summarized the results in Supplementary Tables 1, 2 and Table 1. We presented the quality assessment of the studies with available information in Tables 2, 3.

**Table 1:** Summarizing the results of studies on the effects of fampridine on MS fatigue.

Type of included studies	Reported positive results on MS fatigue	Reported non-significant results on MS fatigue	Comments
Randomized, blinded, placebo-controlled studies	3	10	Total number of studies: 13
Non-randomized, uncontrolled, open-label, or observational studies	19	1	Total number of studies: 20 Out of 20 positive studies, five reported improvement in fatigue only in gait responders.

**Table 2:** Quality assessment of non-randomized studies (ROBINS-I).

Study	Bias due to confounding	Bias in selection of participants into the study	Bias in classification of interventions	Bias due to deviations from intended interventions	Bias due to missing data	Bias in measurement of outcomes	Bias in selection of the reported result	Overall bias
Prugger et al.	Moderate	Moderate	Low	Low	Low	Low	Low	Moderate
Triche et al.	Low	Moderate	Low	Low	Low	Low	Low	Moderate
Korsen et al.	Low	Low	Low	Low	Low	Low	Low	Low
Mitsikostas et al.	Moderate	Low	Low	Low	Moderate	Low	Low	Moderate
Ruck et al.	Low	Low	Low	Low	Low	Low	Low	Low
Bakirtzis et al.	Low	Moderate	Low	Low	Low	Low	Low	Moderate
Rodriguez-Leal et al.	Low	Low	Low	Low	Low	Low	Low	Low
Pavsic et al.	Low	Moderate	Low	Low	Low	Low	Low	Moderate
Allart et al.	Low	Low	Low	Low	Low	Low	Low	Low
Magnin et al./Sagawa et al.	Moderate	Low	Low	Low	Low	Low	Low	Moderate

**Table 3:** Quality assessment of randomized trials (ROB-2).

Study	Randomization process	Deviations from the intended interventions	Missing outcome data	Measurement of the outcome	Selection of the reported result	Overall Bias
Rocca et al.	Low	Low	Low	Some Concerns	Low	Some Concerns
Satchidanand et al.	Some Concerns	Low	Some Concerns	Low	Low	Some Concerns
De Giglio et al.	Low	Low	Low	Low	Low	Some Concerns
Sadeqi et al.	Some Concerns	Low	Low	Some Concerns	Low	Some Concerns
Morrow et al.	Low	Low	Low	Low	Low	Low
Simpson et al.	Low	Low	Low	Low	Low	Low
Pickering et al.	Some Concerns	Low	Some Concerns	Low	Some Concerns	Some Concerns
Valet et al.	Some Concerns	Low	Some Concerns	Low	Low	Some Concerns
Gasperini et al.	Some Concerns	Low	Low	Low	Low	Some Concerns
Zörner et al.	Some Concerns	Low	Some Concerns	Low	Low	Some Concerns
Broicher et al.	Low	Low	Low	Some Concerns	Low	Some Concerns
Goodman et al.	Some Concerns	Low	Low	Some Concerns	Low	Some Concerns
Mavandadi et al.	Low	Low	Low	Low	Low	Low

## Discussion

Fatigue is a common and disabling symptom of MS that leads to poor quality of life (12). The pathophysiology of MS-related fatigue is complex and multifactorial, probably involving inflammation, CNS lesions, altered network recruitment, and metacognitive processes (1, 13–15).

Difficulties in defining and measuring fatigue, lack of objective measures, and the complex pathophysiology of MS-related fatigue have led to difficulty developing effective therapeutics. Substantial evidence supports that non-pharmacological therapies such as cognitive-behavioral therapy (CBT), and exercise positively improve fatigue; however, these modalities are not feasible or available to all patients (16). So, widespread off-label use of medications, such as amphetamine-like and wake-promoting agents, is common among practitioners. A large clinical trial showed that the observed benefit of these medications is primarily due to their placebo effects (17). Finding a medication that targets a pathological mechanism contributing to MS fatigue has remained elusive.

Compounds, such as 4-AP, and its slow-release form, fampridine, improve impulse propagation in demyelinated fibers (18) and have been studied extensively for improving symptoms in MS. Fampridine has also been approved for the treatment of gait dysfunctions in MS. Although, it is unclear to what extent neurophysiological changes caused by demyelination contribute to the subjective sensation of fatigue, it is reasonable to hypothesize that improving signal conduction in demyelinated fibers could improve MS-related fatigue.

In this systematic review, most studies were non-randomized, non-placebo-controlled, open-label studies that reported fatigue scores before and after starting fampridine. Almost all non-randomized studies reported a statistically significant improvement in fatigue scores after starting fampridine compared to the baseline. However, as MS fatigue is highly responsive to a placebo, any intervention can improve fatigue by virtue of its placebo effect. In fact, observing a positive effect in almost all uncontrolled studies is suggestive of the placebo effect of the intervention on MS fatigue. So, non-randomized studies should not contribute to the strength of evidence when assessing an intervention’s effectiveness on MS fatigue.

On the other hand, most randomized, placebo-controlled, blinded studies of fampridine did not report a difference between fampridine and placebo in improving MS fatigue. Only two trials (out of eight) reported a statistically significant difference between fampridine and placebo groups in improving MS fatigue (favoring fampridine). The largest trial, which included patients with impaired processing speed scores at baseline, reported clinically and statistically significant improvement in fatigue compared to placebo. The other randomized, blinded, placebo-controlled study with a positive result had an initial long open-label phase.

The concept of “responders” and “non-responders” has been used to describe people who do and do not experience a certain degree of walking improvement after about 2 weeks of treatment with fampridine (19). It is plausible that there are subgroups of people with MS fatigue who respond better to fampridine. For example, people with impaired processing speed might be a subgroup of patients whose fatigue severity would improve with fampridine (better than placebo). In the fampridine-responder patients, viable, demyelinated axons may contribute heavily to fatigue pathogenesis, while in non-responders, other mechanisms of fatigue predominate.

Two randomized, blinded studies directly evaluated fMRI changes in MS patients receiving fampridine or amantadine. Both reported that these medications produce distinct resting-state functional connectivity (RS FC) alterations within monoaminergic networks—especially in the insular cortex and subcortical regions—and that these changes are linked to reductions in fatigue. However, for fampridine, the association between RS FC alterations and fatigue improvement was only a trend rather than a strong, consistent correlation. Both studies were also constrained by small sample sizes and short follow-up periods. Notably, no significant baseline RS FC differences were detected between treatment groups, and reductions in fatigue occurred across all groups, including placebo (20, 21).

This review represents a systematic and comprehensive literature search focusing on the efficacy of slow-release 4-AP on MS fatigue. This work has several limitations. We did not have access to the required data for a meta-analysis in the majority of the studies. There was considerable heterogeneity among the studies in their design, populations, duration, primary outcome measures, and fatigue instruments, which limits our ability to draw a firm conclusion about the effects of the intervention on MS fatigue. Most studies were non-randomized, uncontrolled studies, which do not contribute in a meaningful way to the overall evidence of the efficacy of fampridine on MS fatigue.

## Conclusion

In conclusion, based on the available data, we cannot currently recommend the off-label use of fampridine to treat MS fatigue. However, we recommend a rigorously designed, randomized, double-blind, placebo-controlled trial, preferably powered to analyze the heterogeneity of treatment effect to find subgroups of patients who respond to this treatment.

## Data Availability

The original contributions presented in the study are included in the article/Supplementary material, further inquiries can be directed to the corresponding author.
