# Supplementary material for: The effects of fampridine on MS-related fatigue: a systematic review
Source: Front Neurol. 2026 Jan 19;16:1720316. doi: 10.3389/fneur.2025.1720316 (PMC12862937; doi:10.3389/fneur.2025.1720316)
Supplement: Supplementary file 1 [file Table_1.docx]

| Author | Year | Country | Study design | Number of participants | Gender | Age | EDSS | Disease duration | MS type | Intervention type | Duration of study | EMIF-SEP | | FSS | | MFIS | | WEIMuS Cognitive | | WEIMuS Physical | | Significant results |
| --- | --- | --- | --- | --- | --- | --- | --- | --- | --- | --- | --- | --- | --- | --- | --- | --- | --- | --- | --- | --- | --- | --- |
|  |  |  |  |  |  |  |  |  |  |  |  | Before | After | Before | After | Before | After | Before | After | Before | After |  |
| Rocca et al. | 2021 | Italy | single-center, prospective, longitudinal, randomized and single-blind trial | 30(15 experiment, 15 healthy control) | Experiment: 6 F, 9 M /control: 10 F, 5 M | Mean (IQR)  Experiment: 44.4 (36–54) /control: 41.3 (32–47) | Median (IQR)  Experiment: 2.0 (1.5–3.0) | Mean (IQR)  Experiment: 12.3 (3.5–20) |  | Fampridine  10 mg twice daily | 4 weeks |  |  |  |  | Experiment: 45.2 ± 13.1) / Control: 46.3 ± 16.1 | Experiment: 34.3 ± 12.1 / Control: 34.4 ± 15.1 |  |  |  |  |  |
| Satchidanand et al. | 2018 | USA | double-blind, placebo-controlled, randomized trial | 61 (45 experiment, 16 | Experiment: 38 F, 7 M /Control: 10 F, 6 M | Experiment: 47.6 ± 10.2 / Control: 53.0 ± 7.5 | Median (range)  Experiment: 3.5 (1.0–6.5) / Control: 3.5 (1.5–6.5) | Experiment: 13.5 ± 7.4 / Control: 13.8 ± 11.9 | Experiment: 37 RR, 7 SP, 1 PP / Control: 12 RR, 4 SP | Dalfampridine  10 mg twice daily | 12 weeks |  |  |  | Mean difference (95% CI)  0.05 (-0.91, 0.80) |  |  |  |  |  |  | Based on FSS, the treatment and placebo groups didn’t have significant differences after intervention. |
| De Giglio et al. | 2019 | Italy | randomized, double-blind, placebo-controlled trial | 120(80 experiment, 40 control) | 74 F, 46 M | 48± 8.2 | Median (range)  4(1-6) | 16±9 | 103 RR, 14 SP, 3 PP | Dalfampridine slow-release  10 mg twice daily | 16 weeks |  |  |  |  | Estimated marginal mean(upper 95% CI)  Experiment: 41.67 (45.36)  / Control: 42.07 (47.08) | Estimated marginal mean(upper 95% CI)  Experiment: week 12: 33.89 (37.71) ; week 16: 37.06 (40.75)  / Control: week 12: 42.33 (48.39); week 16: 40.35 (46.28) |  |  |  |  |  |
| Sadeqi et al. | 2022 | Iran | randomized, double-blind  clinical trial | 54 (27 Dalfampridine, 27 Amantadine) | Dalfampridine: 21 F, 6 M/ Amantadine: 24 F, 3 M | Dalfampridine: 32.93/ Amantadine: 35.37 | Dalfampridine: 1.14/ Amantadine: 1.77 |  | Dalfampridine: 23 RR, 3 CIS, 1 PP/ Amantadine: 20 RR, 3 CIS, 3 SP, 1 PR | Dalfampridine  10 mg twice daily | 8 Weeks |  |  |  |  | Dalfampridine: 47.22 ± 11.71/ Amantadine: 46.74 ± 7.57 | Dalfampridine: month 1: 38.29 ± 15.23; month 2: 34.26 ± 18.30/ Amantadine: month 1: 40.63 ± 14.35; month 2: 36.56 ± 17.12 |  |  |  |  |  |
| Morrow et al. | 2017 | Canada | double-blind, placebo-controlled, randomized, cross-over trial | 60 (Experiment: 29 / Control: 31) | Experiment: 23 F, 6 M/ Control: 23 F, 8 M | Experiment: 46.2 ± 10.7 / Control: 46.7 ± 9.6 | Median (range)  Experiment: 3.5 (1.0–7.0) / Control: 3.0 (1.5–6.5) | Experiment: 11.3 ± 9.6 / Control: 10.0 ± 9.6 | Experiment: 17 RR, 8 SP, 4 PP / Control: 24 RR, 6 SP, 1 PP | Fampridine-SR  10 mg twice daily | 4 weeks |  |  |  |  |  |  |  |  |  |  |  |
| Simpson et al. | 2020 | Australia | randomized double-blind placebo-controlled trial | 40 (20 Experiment, 20 Control ) | Experiment: 12 F, 8 M/ Control: 12 F, 8 M | Median (IQR)  Experiment: 53.5 (47-64)/ Control: 51.5 (43.5-63) |  |  | Experiment: 8 PP, 8 SP, 4 RR/ Control: 10 SP, 5 PP, 5 RR | Fampridine-MR  10 mg twice daily | 12 Weeks |  |  |  |  | Median (IQR)  Experiment: 48.5 (42.5-57.5) | Median (IQR)  Experiment: Week 4: 44 (35-52); Week 8: 43 (36-52) |  |  |  |  |  |
| Pickering et al. | 2017 | Australia | randomised double-blind, placebo-controlled crossover trial | 25 (13 Experiment, 12 Control) | 19 F, 6 M | 54.4 ± 11 |  |  |  | Fampridine  10 mg twice daily | 12 weeks |  |  | 48.3 ± 11.63 | Odds Ratio (90%CI)  Week 4: 1.7 (0.45, 6.25)/ Week 8: 1.9 (0.5–6.96)/ Week 12: 0.8 (0.21–3.11) |  |  |  |  |  |  | Fatigue didn’t have significant improvement in MS patients |
| Valet et al. | 2021 | Belgium | crossover, double-blind trial | 24 | 11 F, 12 M | 46±10 | Median (IQR)  4 (4-5) | Median (IQR)  10 (6-16) | 12 SP, 8 RR, 3 PP | PR-Fampridine  10 mg twice daily | 18 weeks | Median (IQR)  55.2 (42.2–65.35) | Mean effect of Fampridine (95%CI)  − 1.7 (− 12.9;+9.5) |  |  |  |  |  |  |  |  | There wasn’t any significant difference in fatigue score |
| Gasperini et al. | 2016 | USA | Randomized, double-blind,  placebo-controlled trial | 68 Experiment, 64 Control | Experiment: 38 F, 30 M / Control: 33 F, 31 M | Experiment: 49.8 / Control: 49.8 | Mean (range)  Experiment: 5.6 (4-7)/ Control: 5.9 (4-7) |  | Experiment: 31 SP, 24 RR, 13 PP or PR/ Control: 37 SP, 20 RR, 7 PP or PR | PR-Fampridine 10 mg twice daily | 24 weeks |  |  |  |  |  |  |  |  |  |  | “Feeling mentally fatigue” item of MSIS-29 was improved in Fampridine group |
| Zörner et al. | 2016 | Switzerland | Randomized, placebo-controlled, crossover, double-blind trial | 55 | 34 F, 21 M | 48.6±9.8 | 4.9±1.3 | 11.9±7.4 | 29 RR, 21 SP, 5 PP | PR-Fampridine  10 mg twice daily | 18 weeks |  |  |  |  |  |  |  |  |  |  | There is no significant change in fatigue based on WEIMuS |
| Broicher et al. | 2018 | Switzerland | Non-randomized study / Randomized  double-blind, placebo-controlled study | 20 | 10 F, 10 M | 51.4 ± 9.7 | Mean (range)  4.8 (3-6.5) | 11.3 ± 6.6 | 9 RR, 9 SP, 2 PP | PR-Fampridine  10 mg twice daily | 11.5 months |  |  |  |  |  |  | 13.27± 1.75 | 11.32± 2.09 | 18.18± 1.70 | 15.68 ±1.88 |  |
| Goodman et al. | 2007 | USA | Multicenter, randomized, double-blind, placebo-controlled, trial | 36(experiment: 25, control: 11) | experiment: 12 F, 13 M / control: 7 F, 4 M | Mean (range)  experiment: 46(30-61), control: 49.4(36-58) | Mean(range)  Experiment: 5.46(2.5-6.5), control: 5.23(3-6.5) |  | experiment: 18 SP, 4 RR, 3 PP/ control: 8 SP, 3 RR | Fampridine-SR  doses from 10 to 40 mg twice daily, increasing in 5 mg increments weekly | 7 weeks |  |  | Mean (range)  experiment: 5.98(4.1-8.6), control: 6.14(4.4-7) |  | Experiment: 52±16.1 / Control: 50.55±16.4 |  |  |  |  |  | There was a significant decrease of fatigue based on BFI in both treatment and control group; however, this difference between two groups wasn’t significant |
| Mavandadi et al. | 2024 | Iran | randomized, double-blind clinical trial | 77 (42 Experiment, 35 Control) | Experiment: 34 F, 8 M / Control: 28 F, 7 M | Median (range)  Experiment: 39 (35–45) / Control: 38 (30–47) | Median (range)  Experiment: 2.5 (1.5–4) / Control: 2 (1.5–3) | Median (range) month  Experiment: 106 (55.5–169)/ Control: 96 (60–144) | Experiment: 37 RR, 3 SP, 2 PP / Control: 30 RR, 1 SP, 2 PP | Fampridine-ER  10 mg twice daily | 12 weeks |  |  |  |  | Median (range)  Experiment: 43.5 (24.75– 64.5) / Control: 37 (23–50) | Median (range)  Experiment: 39.5 (22.5–56) / Control: 30 (17–46.25) |  |  |  |  |  |

Continuation of Table 1.

| Study ID | MFIS physical | | MFIS cognitive | | MFIS psychological | | WEIMuS total | | BFI | |
| --- | --- | --- | --- | --- | --- | --- | --- | --- | --- | --- |
|  | Before | After | Before | After | Before | After | Before | After | Before | After |
| Rocca et al. | Experiment: 25 ± 6.8 / Control: 22.8 ± 7.3 | Experiment: 18.8 ± 6.7/ Control: 16.6 ± 7.2 | Experiment: 16.7 ± 8.7 / Control: 19.1 ± 9.4 | Experiment: 12.7 ± 7.8 / Control: 14.6 ± 9.2 | Experiment: 3.6 ± 1.4 / Control: 4.3 ± 1.4 | Experiment: 2.8 ± 1.6 / Control: 3.1 ± 2.2 |  |  |  |  |
| De Giglio et al. | Estimated marginal mean(upper 95% CI)  Experiment: 19.51(21.49)  / Control: 21.36(23.73) | Estimated marginal mean(upper 95% CI)  Experiment: week 12: 17.01(18.98); week 16: 18.46(20.44)  / Control: week 12: 21.23(24.26); week 16: 20.44(23.07) | Estimated marginal mean(upper 95% CI)  Experiment: 17.93(20.04)  / Control: 16.48(19.38) | Estimated marginal mean(upper 95% CI)  Experiment: week 12: 13.45(15.42); week 16: 14.90(16.87)  / Control: week 12: 16.74(19.64); week 16: 15.95(18.98) | Estimated marginal mean(upper 95% CI)  Experiment: 3.69(4.22)  / Control: 3.82(4.48) | Estimated marginal mean(upper 95% CI)  Experiment: week 12: 3.03(3.69); week 16: 3.29(3.82)  / Control: week 12: 4.08(4.87); week 16: 3.95 (4.74) |  |  |  |  |
| Broicher et al. |  |  |  |  |  |  | Mean (SEM)  31.7 (2.98) | Mean (SEM)  27.17 ± 3.34 |  |  |
| Goodman et al. |  |  |  |  |  |  |  |  | Experiment: 6.13±1.6/ Control: 6.43±2.2 | Change in score (mean (SEM))  Experiment: Week 1: -0.19 (0.12), Week 2: -0.31 (0.16), Week 3: -0.34 (0.16), Week 4: -0.39 (0.19), Week 5: -0.66 (0.18), Week 6: -0.68 (0.25), Week 7: -0.81 (0.23)/  Control: Week 1: -0.21 (0.31), Week 2: -0.64 (0.44), Week 3: -0.80 (0.55), Week 4: -1.08 (0.56), Week 5: -1.50 (0.73), Week 6: -1.56 (0.65), Week 7: -1.84 (0.75) |
| Mavandadi et al. | Median (range)  Experiment: 20 (12.75–29.25)/ Control: 18 (17–23) | Median (range)  Experiment: 16 (10–22.25)/ Control: 14.5 (8–19.25) | Median (range)  Experiment: 18 (11.75–32.5)/ Control: 19 (9–24) | Median (range)  Experiment: 17 (11–27.75)/ Control: 15 (8–20) | Median (range)  Experiment: 3 (1–7)/ Control: 3 (1–5) | Median (range)  Experiment: 2.5 (1–5)/ Control: 4 (1–6) |  |  |  |  |

Continuation of table 1
