# Supplementary material for: The effects of fampridine on MS-related fatigue: a systematic review
Source: Front Neurol. 2026 Jan 19;16:1720316. doi: 10.3389/fneur.2025.1720316 (PMC12862937; doi:10.3389/fneur.2025.1720316)
Supplement: Supplementary file 2 [file Table_2.docx]

| Author | Year | Country | Study design | Number of participants | Gender | Age | EDSS | Disease duration | MS type | Intervention type | Duration of study | EMIF-SEP | | FSS | | F-VAS | | | MFIS | | WEIMuS Cognitive | | WEIMuS Physical | | Significant results |
| --- | --- | --- | --- | --- | --- | --- | --- | --- | --- | --- | --- | --- | --- | --- | --- | --- | --- | --- | --- | --- | --- | --- | --- | --- | --- |
|  |  |  |  |  |  |  |  |  |  |  |  | Before | After | Before | After | Before | After | | Before | After | Before | After | Before | After |  |
| Prugger et al. | 2013 | Austria | Case series | 67 | 46 F, 21 M | 47.8 ± 8.4 | 4.8 ± 1.6 | 16.5 ± 7.3 | 34 SP, 17 RR, 16 PP | PR-Fampridine  10 mg twice daily | 4 weeks |  |  | 5.2 ± 1.4 | 4.7 ± 1.5 |  |  | |  |  |  |  |  |  |  |
| Korsen et al. | 2016 | Germany | prospective, single center, single arm, observational study | 34(responder 22, non-responder 12) | 22 F, 12 M | Responder: 48.0 ± 10.4 / non-responder: 48.4 ± 7.8 | Median (IQR)  Responder: 4.0 (4.0–5.0) / non-responder: 4.5 (4.0–6.5) |  | RR: 18, SP: 12, PP: 4 | Dalfampridine  10 mg twice daily | 2 weeks |  |  | 49.3 ± 12.1 | 48.1 ± 13.2 |  |  | |  |  |  |  |  |  |  |
| Guyot et al. | 2014 | France | retrospective study | 104 |  | 54 ± 18.6 | Median 6 |  |  | Dalfampridine | 6 months | 57.6 ± 19.5% | 37.8 ± 19% |  |  |  |  | |  |  |  |  |  |  |  |
| Di Sapio et al. | 2018 | Italy | Nonrandomized study | 10 |  |  |  |  |  | Fampridine | 2 weeks |  |  |  |  |  |  | |  |  |  |  |  |  | The responder patients had reduction in fatigue based on FSS score ; however, MFIS improved only in 1 patient |
| Mitsikostas et al. | 2021 | Greece | Cohort study | 102 | 59 F, 43 M | 47.78 ±10.852 | 4.7 ±0.88 | 11.44 ±7.828 | 81 PP, 8 SP, 7 PP, 4 PR, 2 CIS | PR-Fampridine | 24 Weeks |  |  |  |  |  |  | | 36.68 ± 16.981 | Week 12: 34.45 ±17.272/ week 24: 32.77 ±16.123 |  |  |  |  |  |
| Barros et al. | 2016 | Portugal | Prospective study | 20(12 responder, 8 non-responder) | 13 F, 7 M | Median 53 | Median 6 |  |  | Fampridine | 2 weeks |  |  | Median  Responder: 39 / Non-responder: 37.5 | Median  Responder: 46 / Non-responder: 40.5 |  | |  |  |  |  |  |  |  | The Responder group had improvement in fatigue in contrast to the non-responder group |
| Kobelt et al. | 2018 | Sweden | Cross sectional | 5105(934 experiment, 4171 control) | Experiment: 80% F, 20 % M / Control: 83% F, 17% M | Experiment: 53.3 , Control: 54.5 | Median  Experiment: 6 / Control: 5 |  |  | Fampridine-PR |  |  |  |  |  |  | |  |  |  |  |  |  |  | The treatment group had lower fatigue score in comparison with control group (VAS) |
| Berkovich et al. | 2016 | USA | Observational study | 11 | 11 F |  |  |  |  | Fampridine-ER  10 mg twice daily | 8 weeks |  |  |  |  |  | |  |  |  |  |  |  |  | Fatigue component of MS quality of life inventory was improved. |
| Ruck et al. | 2014 | Germany | Non randomized study | 52 | 34 F, 18 M | 50 ± 0.17 | 5.3 ± 0.02 |  | 25 SP, 16 PP, 11 RR | Dalfampridine | 12 months |  |  |  |  |  | |  |  |  |  |  |  |  |  |
| Rodriguez-Leal et al. | 2016 | Germany | Non-randomized study | 134 | 87 F, 46 M | 54.3± 11.4 | 5.2 ± 1.3 | 13.5 ±8.4 | 55 RR, 40 SP, 36 PP | Fampridine |  |  |  |  |  |  | |  |  |  |  |  | 16.5 ±7.1 | Week 2: 12.9 ± 7.2  Year 1: 15.3 ± 7.2  Year 2: 15.3 ±6.8 |  |
| Rodriguez-Leal et al. | 2019 | Germany | Non randomized trial | 189 | 122 F, 67 M | 53.55 ± 10.83 | 5.22 ± 1.29 | 12.92 ± 10.83 | 77 RR, 61 SP, 50 PP | Fampridine | 2 weeks |  |  |  |  |  | |  |  |  | 12.63 ± 8.06 | 9.61 ± 8.41 | 17.18 ± 7.53 | 13.99 ± 8.04 |  |
| Rodriguez et al. | 2022 | Dominican republic | Prospective observational study | 33 |  |  |  |  |  | Fampridine-ER | 4 weeks |  |  |  |  |  | |  |  |  |  |  |  |  | The patients had improvement in fatigue |
| Magnin et al. | 2015 | France | prospective monocentric open label trial | 50 | 34 F, 16 M | 51.4 ± 11.7 | 5.3 ± 1.1 | 14.3 ± 9.3 | 16 PP, 24 SP, 10 RR | Fampridine | 3 weeks |  |  |  |  | 50.56 ± 20.65 | | Week 2: 38.1 ± 22.22/ week 3: 36.62 ± 23.82 |  |  |  |  |  |  |  |
| Kurtuncu et al. | 2016 | Turkey | Retrospective study | 179 | 109 F, 70 M |  | 5.8± 0.8 |  |  | Fampridine-ER |  |  |  |  |  |  | |  |  |  |  |  |  |  | In the study, 49.2% of patients reported improvement in fatigue |
| Farrell et al. | 2014 | UK | Non-randomized study | 76 | 46 F, 30 M | 55 | 6.3 |  |  | PR-Fampridine  10 mg twice daily | 4 Weeks |  |  |  |  |  | |  |  |  |  |  |  |  | Patients had improvement in fatigue. |
| Triche et al. | 2016 | USA | single-site observational study | 31 | 24 F, 7 M | 53.7 ± 10.3 | 5.1 ± 1.7 | 13.1 ± 8.8 | 25 RR, 4 SP, 2 PP | Dalfampridine-ER | 14 weeks |  |  |  |  |  | |  |  |  |  |  |  |  |  |
| Korsen et al. | 2016 | Germany | prospective, single center, single arm, observational study | Responders: 22 | Responders: 15 F, 7 M | Responders: 48.0 ± 10.4 | Median (IQR)  Responders: 4.0 (4.0–5.0) |  | Responders: 13 RR, 8 SP, 1 PP | Dalfampridine  10 mg twice daily | 2 weeks |  |  | Responders: 48.8 ± 11.7 | Responders: 45.2 ± 13.5 |  | |  |  |  |  |  |  |  |  |
| Sagawa et al. | 2016 | France | Non-randomized study | 50 | 34 F, 16 M | 51.4 ± 11.7 | 5.2 ± 1.1 | 14.3 ± 9.3 | 24 SP, 16 PP, 11 RR | Fampridine  10 mg twice daily | 3 weeks |  |  |  |  |  | |  |  |  |  |  |  |  |  |
| Bakirtzis et al. | 2018 | Greece | observational, open label study | 54( responders: 35, non-responders: 19) | Responders: 17 F, 18 M / non-responders: 11 F, 8 M | Responders: 52.4 / non-responders: 48 | Responders: 5.3/ non-responders: 5.9 | Responders: 14.4/ non-responders: 13.2 | Responders: 11 RR, 11 PP, 13 SP / non-responders: 7 RR, 2 PP, 10 SP |  | 48 weeks |  |  |  |  |  | |  | Responders: 43.9± 14.4 / non-responders: 43.3 ± 13.8 | Responders: week 24: 42.7± 13.7, week 48(n=33): 41.2± 13.2 / non-responders: week 24: 43.6± 13, week 48: 41.2± 14.6 |  |  |  |  |  |
| Pavsic et al. | 2015 | Slovenia | prospective non-randomized study | Responders: 17 |  |  |  |  |  | Fampridine  10 mg twice daily | 4 weeks |  |  |  |  |  | |  | Responders: 51.2 ± 17.4 | Responders: 39.3 ± 13.2 |  |  |  |  |  |
| Allart et al. | 2015 | France | Non randomized trial | 112 (responders: 83 / non-responders: 29) | Responders: 44 F, 39 M / non-responders: 18 F, 11 M | Responders: 50.7 ± 11 / non-responders: 54.9 ± 12.8 | Median (IQR)  Responders: 6 (2) / non-responders: 5.5 (2.5) | Responders: 16.7 ± 9 / non-responders: 21.4 ± 13 | Responders: 24 RR, 35 SP, 24 PP / non-responders: 11 RR, 10 SP, 8 PP | Fampridine-SR  10 mg twice daily | 3 months |  |  | Responders: 5.6 ± 1.1 / non-responders: 5.4 ± 1.5 | Responders: Week2: 4.6±1.4  Month 3: 4.7±1.4 / non-responders: week 2: 5.5 ± 1.5 | Responders: 56.1 ± 20.3 / non-responders: 56.6 ± 23.1 | | Responders: Week2: 41.4 ± 22.3  Month 3: 48.8 ± 20.9 / non-responders: week2: 60.3 ± 24.2 |  |  |  |  |  |  |  |

Continuation of table 2

| Study ID | Fatigability index | | Fatigue subscale of performance scale | | Fatigue subscale of PERSEPP | | MFIS physical | | MFIS cognitive | | MFIS psychological | | FSMC-motor | | FSMC-cognitive | |
| --- | --- | --- | --- | --- | --- | --- | --- | --- | --- | --- | --- | --- | --- | --- | --- | --- |
|  | Before | After | Before | After | Before | After | Before | After | Before | After | Before | After | Before | After | Before | After |
| Triche et al. |  |  | Median (IQR)  3.0 (2.0, 4.0) | Median (IQR)  3.0 (2.0, 4.0) |  |  |  |  |  |  |  |  |  |  |  |  |
| Magnin et al. |  |  |  |  | 44 (29.2) | Week 2: 52.4 ± 32.8 /Week 3: 52.8 ± 34.7 |  |  |  |  |  |  |  |  |  |  |
| Allart et al. | Responders: -20.3 ± 20.5  / non-responders: -17.2 ± 18.2 | Responders: Week2: -19±14.2  Month 3: -15.8±14.8 / non-responders: week 2: -15.5 ± 17.3 |  |  |  |  |  |  |  |  |  |  |  |  |  |  |
| Ruck et al. |  |  |  |  |  |  | Responders: 24.2± 6.1 / non-responders: 24.3± 7 | Responders: week 24: 24.5± 6.3, week 48(n=33): 23.8± 6.4 / non-responders: week 24: 25.7± 6.6, week 48: 24.8± 7.6 | Responders: 14.8± 8.8 / non-responders: 13.1± 7.2 | Responders: week 24: 14.2± 7.9, week 48(n=33): 12.9± 7.2 / non-responders: week 24: 13.1± 5.8, week 48: 11.2± 7 | Responders: 4.6± 1.6 / non-responders: 5.3± 1.8 | Responders: week 24: 4.8± 1.9, week 48(n=33): 4.6± 1.6 / non-responders: week 24: 4.8± 1.4, week 48: 5.1± 1.5 | 38.98 | Week 2: 34.32, month 12: 34.23 | 31.78 | Week 2: 27.56, month 12: 27.31 |
| Bakirtzis et al. |  |  |  |  |  |  | 27.8 ± 5.6 | 22.2 ± 7.0 | 17.9 ± 11.8 | 12.5 ± 8.0 | 5.5 ± 2.1 | 4.6 ± 1.9 |  |  |  |  |
| Pavsic et al. |  |  |  |  |  |  | Responders: 24.2± 6.1 / non-responders: 24.3± 7 | Responders: week 24: 24.5± 6.3, week 48(n=33): 23.8± 6.4 / non-responders: week 24: 25.7± 6.6, week 48: 24.8± 7.6 | Responders: 14.8± 8.8 / non-responders: 13.1± 7.2 | Responders: week 24: 14.2± 7.9, week 48(n=33): 12.9± 7.2 / non-responders: week 24: 13.1± 5.8, week 48: 11.2± 7 | Responders: 4.6± 1.6 / non-responders: 5.3± 1.8 | Responders: week 24: 4.8± 1.9, week 48(n=33): 4.6± 1.6 / non-responders: week 24: 4.8± 1.4, week 48: 5.1± 1.5 |  |  |  |  |
